# Delineating Bacteriostatic and Bactericidal Targets in Mycobacteria Using IPTG Inducible Antisense Expression

**DOI:** 10.1371/journal.pone.0005923

**Published:** 2009-06-15

**Authors:** Parvinder Kaur, Saurabh Agarwal, Santanu Datta

**Affiliations:** AstraZeneca India Pvt. Ltd. Hebbal, Bangalore, India; University of Hyderabad, India

## Abstract

In order to identify novel high value antibacterial targets it is desirable to delineate whether the inactivation of the target enzyme will lead to bacterial death or stasis. This knowledge is particularly important in slow growing organisms, like mycobacteria, where most of the viable anti-tubercular agents are bactericidal. A bactericidal target can be identified through the conditional deletion or inactivation of the target gene at a relatively high cell number and subsequently following the time course of survival for the bacteria. A simple protocol to execute conditional inactivation of a gene is by antisense expression. We have developed a mycobacteria specific IPTG inducible vector system and monitored the effect of antisense inhibition of several known essential genes in mycobacteria by following their survival kinetics. By this method, we could differentiate between genes whose down regulation lead to bacteriostatic or bactericidal effect. Targets for standard anti-tubercular drugs like *inh*A for isoniazid, *rpo*B and C for rifampicin, and *gyr* A/B for flouroquinolones were shown to be bactericidal. In contrast targets like *Fts*Z behaved in a bacteriostatic manner. Induction of antisense expression in *emb*B and ribosomal RNA genes, viz., *rpl*J and *rps*L showed only a marginal growth inhibition. The specificity of the antisense inhibition was conclusively shown in the case of auxotrophic gene *ilv*B. The bactericidal activity following antisense expression of *ilv*B was completely reversed when the growth media was supplemented with the isoleucine, leucine, valine and pantothenate. Additionally, under these conditions the expression of several genes in branched chain amino acid pathway was severely suppressed indicating targeted gene inactivation.

## Introduction

Though the genome sequence of various infectious pathogens including *M. tuberculosis* has been available for over a decade [1], no new antibiotics have come to market that target a novel gene product. Even the pharmaceutical industry pipeline in the pre-clinical and clinical phase has only a few candidate molecules that interact with enzyme targets identified and validated by genomic techniques [2]. At a first glance this setback looks counterintuitive. Literature and various bioinformatics websites are replete with data on the essential genes of various microorganisms including mycobacteria [3], [4]. In addition to classical knockout data, we now have various high throughput techniques like transposon mutagenesis that have been successfully used to identify essential genes [5], [6]. Then, why is there a drought of new drugs or leads that are based on the genomic paradigm? One of the reasons we argue is the over reliance on essentiality as a genetic requirement for a valid anti-infective target. Essentiality of a gene is only a necessary genetic requirement for an anti-infective target but not a sufficient one.

A valid anti-infective drug target is one whose inhibition leads to the death of bacteria. However, the usual practice to establish essentiality of a gene is to monitor growth of the bacteria in the absence of the target gene. This is conceptually similar to the test done for the susceptibility or the MIC (minimum inhibitory concentration) measurement of antimicrobial agents. Under this experimental setup both bacteriostatic and bactericidal targets behave in a similar fashion. In order to delineate static and cidal targets one could gradually reduce the level of target gene expression and monitor the survival kinetics of the bacteria. This can be done either by putting the target gene under the control of an inducible promoter, or by gradually reducing the wild-type gene expression by antisense inhibition. In the former, there is a chromosomal insertion of the inducible promoter upstream of the target gene, while in the latter case; constructs are made in compatible plasmids such that the gene is placed in antisense orientation under an inducible promoter. In the antisense mode, upon induction, the expression of the gene is repressed as the expression of the antisense RNA increases. Under saturating expression of the antisense RNA, the target inhibition might be equivalent to a classical knockout of the gene. Two pharmaceutical companies, GSK and Elitra have reported the use of antisense technology across the genome to identify essential genes in the pathogen *Staphylococcus aureus* [7], [8]. However bacteriostatic and bactericidal targets were not differentiated.

In most anti-infective drug discovery cascade bactericidal effect of a compound is rarely monitored. This practice looks minimalist in view of the mounting evidence that in most cases bactericidal drug outperforms bacteriostatic drugs. This lacuna has been pointed out by Stratton, who opined for the use of bactericidal antibiotics to counter drug resistance [9]. In addition to *M. tuberculosis*, in diseases like endocarditis, clinical efficacy is found only with bactericidal drugs. It is also argued by Levinson that for bacteriostatic drugs to have efficacy it is necessary that it be augmented by host defences to clear tissues of the infecting microorganism [10]. With dysfunctional immune system the residual pathogen resumes growth after the withdrawal of the bacteriostatic drug and the infection relapses. As a consequence it would seem logical to identify targets especially in mycobacteria whose inhibition would be bactericidal. These then would be the choicest targets for anti-tubercular drug discovery.

Since the *Lac* operon is absent in mycobacteria, an IPTG inducible system was imported from *E. coli* as a plasmid based system for its operation in mycobacteria. Several target genes were cloned in the antisense orientation and the downstream effects viz., bactericidal or bacteriostatic, were monitored by its survival kinetics following induction of antisense expression. Using this technology we could delineate targets whose down regulation or inhibition would be cidal or static for the organism.

## Results

### Repressor Operator Interaction

Development of a lac inducible system with a linear response that is operational in mycobacteria requires the understanding of the basics of repressor operator interaction. Due to the absence of the lac operon – and more specifically the permease *lac*Y - it could be speculated that in mycobacteria, the inducer would enter the cell by a diffusion-controlled fashion [11].

**Figure 1:**
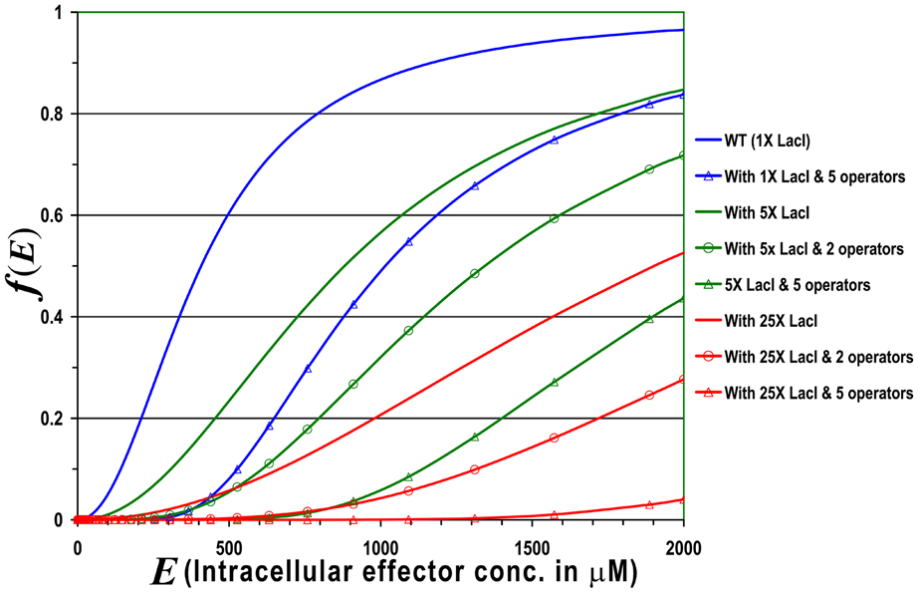
Number of operators *vs. Lac*I expression. Plot of fraction of free Lac operators as a function of effector concentration for different amounts of intracellular *Lac*I and different number of operators. Here 1X is considered to be the intracellular concentration of *Lac*I in wild type *E.coli.*

The semi-quantitative analysis of operator–repressor interaction (detailed in the supporting information File S1) was used to identify conditions that would give a minimal leaky expression while introducing an operationally linear induction profile. Since the parameters that could be varied are the levels of *Lac* I and the number of tandem operators, we plotted *f* (*E*) as a function of these two variables using equations 3 and 5. The values of *K*_1_ as 0.0252 (μM)^−2^, n as 2.09 and *K* as 7200 from the results of Yagil and Yagil [12] were used in the simulation. The results (Figure 1) indicate that with one operator we get close to linear induction and low level of leaky expression when there is about in 5 to 25-fold excess of *Lac*I expression over its normal level. With multiple operators though the basal level expression is severely repressed, the induction becomes non-linear and the maximum induction is not reached. We opined that using a single operator in conjunction with a constitutive promoter of moderate strength for *LacI* would result in linear induction with a negligible uninduced basal level expression. For ease of operation, an additional feature of this constitutive promoter should be that it is equally functional in both the mycobacterial species, *M. smegamtis* and *M. tuberculosis*.

### Construction of the lac-inducible mycobacterial vector pAZI9018b

The inducible expression vector pAZI9018b has the following major elements: IPTG inducible reporter gene, a constitutively expressing *Lac*I gene, an antibiotic marker gene and ori sequences aiding the plasmid stability, integrity and replication in mycobacterial and *E. coli* background. For designing the inducible system, the *Lac* operator was cloned downstream to the promoter and upstream to the reporter gene *Lac*Z. Regarding the choice of the specific promoters that will be suitable in mycobacteria we used the mycobacterial consensus sigA promoter sequence that was identified by Unniraman *et al*. [13] as TTGACA/T(-35 region)-17 bp spacer -TATAA/CT(-10 region)-7 bp-G/A as compared to that of *E. coli* TTGACA(-35 region)-17 bp spacer -TATAAT(-10 region)-7 bp-A. It was seen that the pTrc promoter (ttgacaattaatcat ccggctcgtataatgtgtggAa) from the plasmid p*Trc*99A matched suitably with the consensus housekeeping mycobacterial *Sig*A sequence. This strategy would be useful in using the vector in future experiments under *in-vivo* conditions.

**Figure 2:**
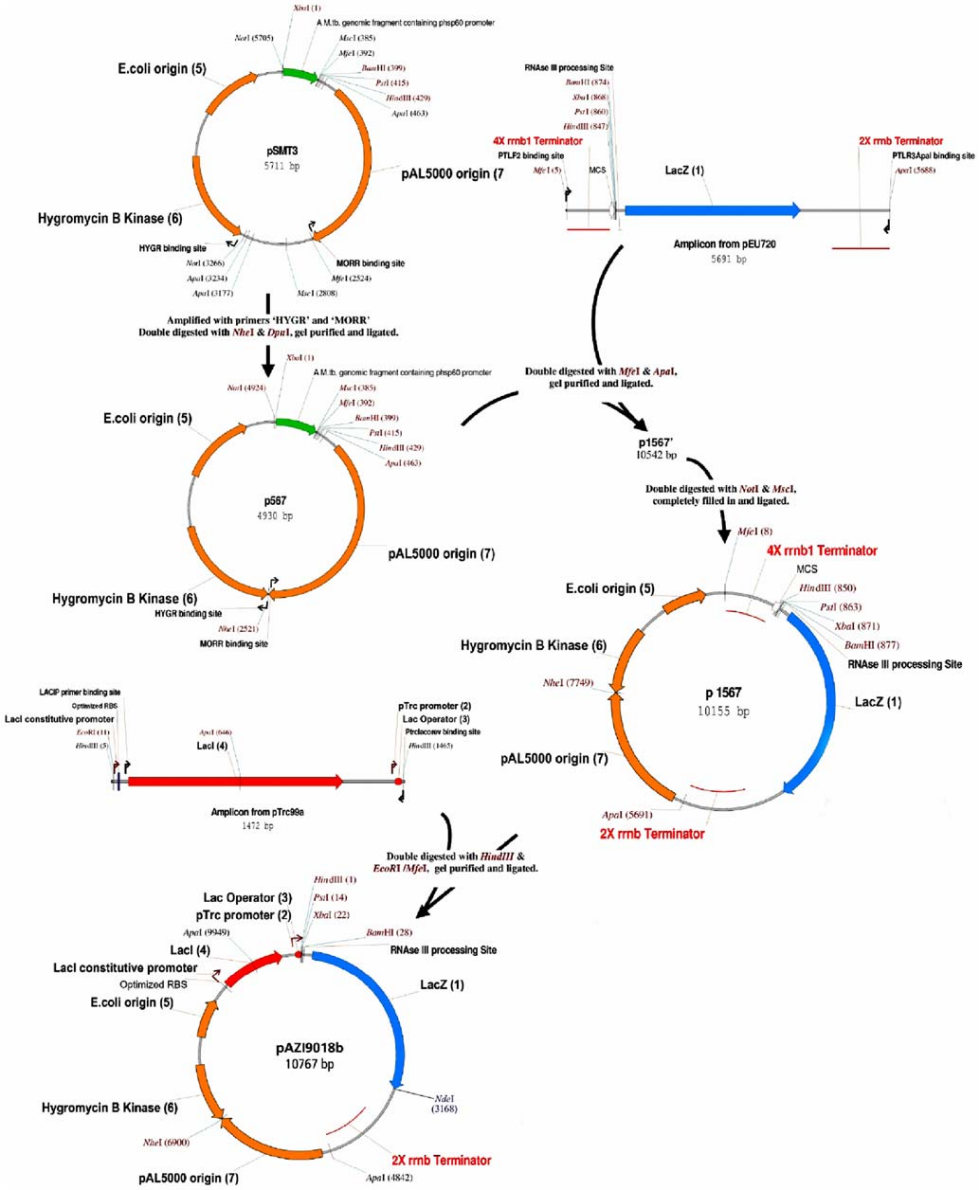
Construction of pAZI9018b (10767 bp). The genes for selection marker hygromycin, the origins of replication for *E. coli* (*Ori*E) and mycobacteria (*Ori*M) were taken from pSMT3 a shuttle vector. Reporter gene *LacZ* and the rrnB terminators were taken from a plasmid pEU720. Constitutive *LacI*, mycobacterial SigA promoter and lac operator genes were derived from p*Trc*99A. This inducible vector has 7 elements: 1) *LacZ* reporter gene, 2) p*Trc* promoter, 3) *Lac* operator, 4) Constitutive *LacI*, 5) *E.coli* origin of replication, 6) Hygromycin B Kinase, the selection marker, 7) p*AL5000* origin- mycobacterial origin of replication genes.

Regarding the constitutive lac repressor, we argued that to get a close to linear induction profile and a low basal expression of the target gene in mycobacteria, we needed a few fold higher expression of Lac repressor in mycobacteria as compared to that present in wild type *E. coli.* Since *Lac*I is present in low amounts in *E. coli* (∼10 tetramers/cell) [14], a moderate promoter when present in a plasmid with a low copy number (∼3–4 per cell) should be able to produce the required amount of lac repressor in mycobacteria. Additionally it would be advantageous if the expression level of the promoter would remain unaltered when moved from the fast growing *M. smegmatis* to the slow growing *M. tuberculosis* and *M. bovis* BCG. The T150 promoter sequence as described by Bashyam *et.al*. [15] was chosen as the constitutive promoter for the lac repressor as it was of moderate strength and had been shown to be equipotent in both the mycobacterial species. It had an additional advantage that its sequence (TTGACA CTTTGCGACACGCTTTTATCAT ) had a high similarity with that of mycobacterial sigA promoter ensuring that it would be driven by the mycobacterial house-keeping sigma factor and thus would be present nearly at all times of growth phase. The restriction sites *Bam*HI and *Nde*I in the vector are suitably positioned so that any foreign gene could be inserted in to the vector replacing the reporter *LacZ* (Figure 2).

### IPTG regulated β-galactosidase activity in mycobacteria

**Figure 3:**
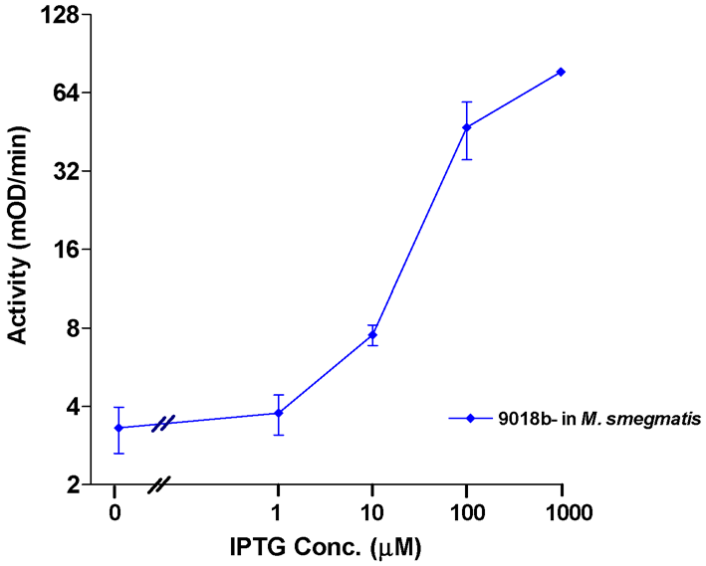
Validation of the inducible promoter system by β-galactosidase assay. *Mycobacterium smegmatis* mc^2^155 cultures harboring the plasmid pAZI 9018b were induced in triplicate with IPTG in a dose dependent manner (0,1,10,100,1000 μM). The *β*-galactosidase activity was monitored at O.D.410 in SpectraMax Plus^384^ spectrophotometer at 37°C after adding ONPG.

The β-galactosidase activity of *M. smegmatis* harboring the vector pAZI9018b was monitored. The assay revealed a linear increase in the enzyme activity (mO.D._410 nm_/min) over a wide range of IPTG concentration (Figure 3). The upregulation of expression following induction was about 30 fold. The level of leaky expression was low as there was a fairly tight regulation at zero IPTG concentration. The results indicated that the constitutive promoter T150 produced enough repressor so as to block the transcription of the *lacZ* gene in the absence of any inducer. It was also observed with times of induction less than four hours the β-galactosidase activity reduced considerably and with induction time greater than 12 hours the fold induction reduced as there was an accumulation of leaky expression (data not shown). The results indicate that it took about a generation time (4-hours) in *M. smegmatis* for observing the optimized effect of the IPTG induction. It was also observed that various levels of IPTG did not have any effect on the growth rate of the host bacteria (data not shown).

### Conditional knockdown (KD) of *Fts*Z by antisense expression mRNA in *M. smegmatis*

**Figure 4:**
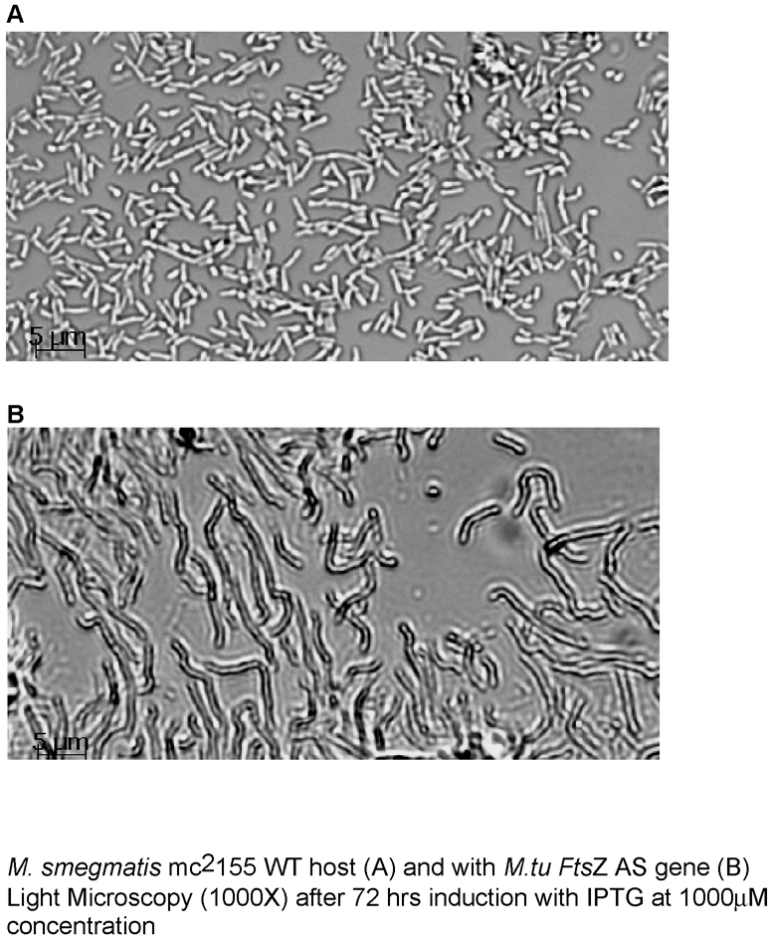
*Fts*Z antisense expression in mycobacteria. Microscopic (1000x) observation of *M. tuberculosis Fts*Z antisense construct in *M. smegmatis* mc^2^155: *M. smegmatis* mc^2^155 transformed with Mtu *Fts*Z antisense (AS) construct. The colonies appeared after 8 days (*vs*.control in 3 days) & showed poor growth in broth (inhibited). Culture concentrated to normalize the cell number (vs. WT) for induction with IPTG. Panel A. *M. smegmatis* mc^2^155 host (WT) and Panel B. *M. smegmatis* mc^2^155 with Mtu *Fts*Z antisense gene, both induced for 48 hrs. with 1 mM IPTG. The induced cultures of *Fts*Z antisense clone (Panel B) showed filamentation with elongated cells (no septation), as observed under phase contrast conditions in 1000X. Micrographs were taken using AxioVision Rel. 4.5 software.

**Figure 5:**
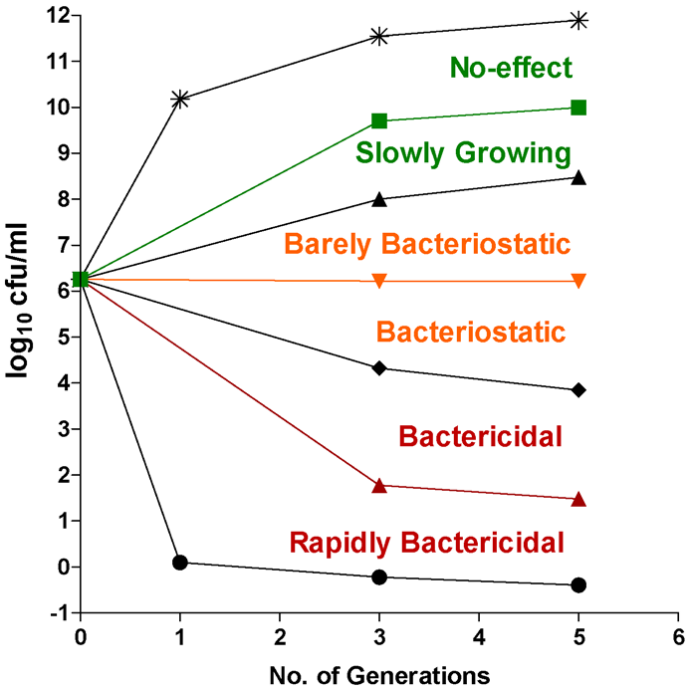
Bacteriostatic and bactericidal target zones. Generic survival kinetics on delineating bactericidal and bacteriostatic targets. The zones differentiating cidality, stasis and no effect are indicated.

**Figure 6:**
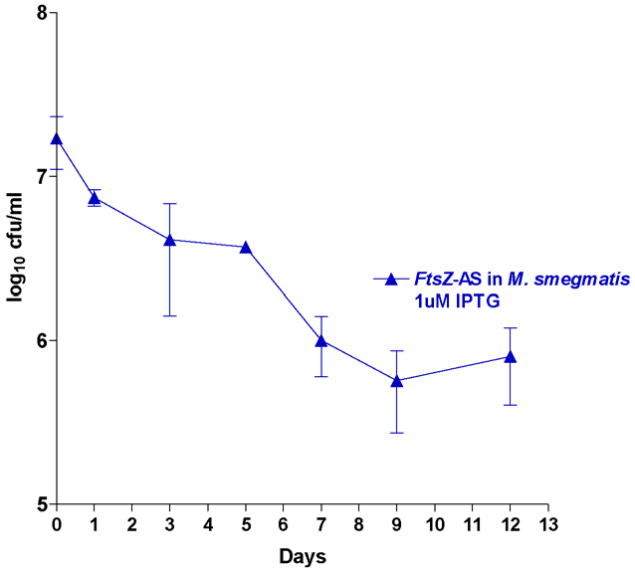
Survival kinetics of *M.smegmatis* (harbouring *M. tuberculosis Fts*Z-AS plasmid). Induction at 1 uM IPTG of the *Fts*Z *r-M. smegmatis* shows ∼1.5 log10 cfu reduction, categorizing it as a bacteriostatic target.

*Fts*Z protein forms a ring at the site of bacterial cell division. Over or under production of this protein causes an imbalance in the cell division and results in cell lysis or filamentation respectively [16], [17]. We down-regulated *FtsZ* gene by cloning it in antisense orientation replacing the *LacZ* in pAZI9018b. Though it was the *M. tuberculosis FtsZ* gene that had been cloned in antisense orientation, it could down regulate the *M. smegmatis Fts*Z expression, as there was about 92% sequence identity at the nucleotide level. Compared to the wild type strain, the induced antisense *Fts*Z strain showed a high degree of filamentation (Figure 4). This observation is in accordance to the earlier observation that deficiency of *Fts*Z causes filamentation in mycobacteria [18], proving that the antisense mRNA was capable of inhibiting the expression of the genomic driven *FtsZ* sense gene. In order to delineate whether the downstream effect of a target downregulation is bacteriostatic or bactericidal one has to define a mycobacterial benchmark. This is illustrated in the generic mycobacterial survival kinetics (Figure 5) where various zones have been defined. Though, the standard 99.9% kill is defined as bactericidal for antibiotics, the slow growth of *M. tuberculosis* brings in an element of time dependence while evaluating cidality and stasis. From the survival kinetics of *Fts*Z antisense strain it was seen that the target behaved in a bacteriostatic fashion. There was a meagre drop of 1.5 log_10_ in cfu at an inducer concentration of 10 µM IPTG (Figure 6). There was no further reduction in cfu at higher IPTG concentration.

### Conditional KD of *gyr*A and *gyr*B in *M. smegmatis* and *M. tuberculosis*

Gyrase is an essential enzyme containing two subunits encoded by the genes *gyr*A and *gyr*B. It is the target of the bactericidal antibiotics belonging to the class of quinolones and coumarins that bind to *gyr*A and *gyr*B respectively. Its essentiality is derived from the fact that it is the only enzyme that is able to introduce negative supercoils into DNA which is critical in cellular processes like replication, recombination and transcription that depend on the unwinding of DNA strands.

**Figure 7:**
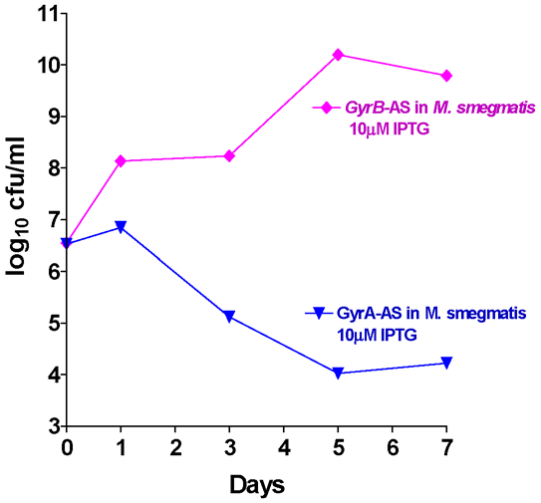
Survival kinetics of *M. smegmatis* (with *M. tuberculosis gyr*A-AS and *gyrB* –AS plasmids individually). Induction at 10 uM IPTG concentration. In the case of *gyr*A-AS r-*M. smegmatis* the cfu reduction is >3 log10 (bactericidal target), but *gyr*B-AS showed no cfu reduction as the orphan gyrB takes over once the other gyrB transcript is blocked.

**Figure 8:**
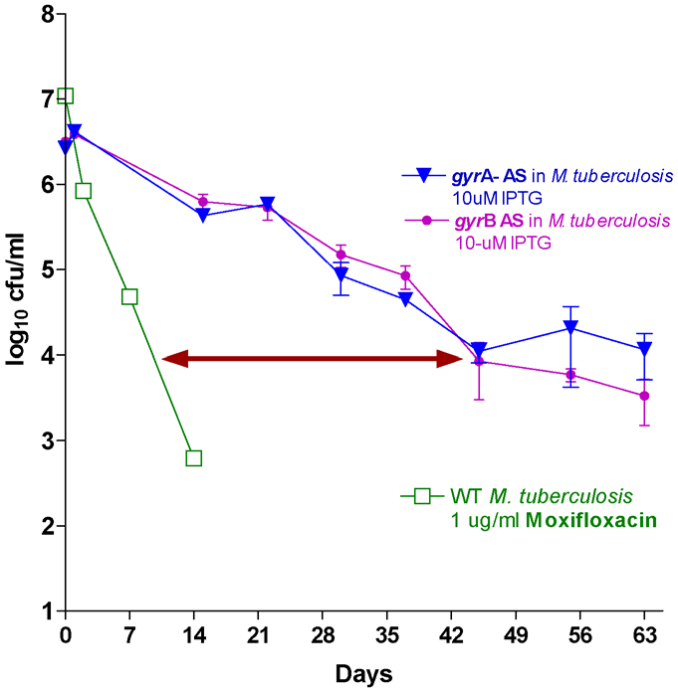
Survival kinetics of *M. tuberculosis* (with *M. tuberculosis gyr*A-AS and *gyrB*-AS plasmids individually). Induction at 10 uM IPTG. *gyr*A-AS is bactericidal whereas *gyrB*-AS is showing no-effect due to an extra copy of *gyr*B gene (orphan *gyr*B). Survival kinetics of wild type *M. tuberculosis* upon exposure to 1 ug/ml conc. of Moxifloxacin is cidal.

Full-length antisense genes of *gyr*A and *gyr*B were cloned into pAZI9018b and transformed into *M. smegmatis* and the survival kinetics following the antisense induction of the individual genes monitored. The results (Figure 7) indicate that while induction of antisense of *gyr*A resulted in more that 3-log_10_ reduction of the bacteria, the induction of antisense of *gyr*B was well tolerated and the bacteria grew normally. This data looked contradictory at the outset. However during the literature search it was seen that a gene designated as orphan *gyr*B was identified in *M. smegmatis* [19]. We speculated that while the wild type *gyr*B expression is reduced the orphan *gyr*B was capable in taking over the activity and thus preventing cell death. In order to prove this hypothesis we transformed the same constructs in *M. tuberculosis* and monitored the survival kinetics. The global analysis of *M. tuberculosis* genome did not indicate the presence of any additional putative *gyr*B; also the tranposon mutagens data indicated that both these genes were essential in *M. tuberculosis* [6]. The survival kinetics data (Figure 8) indicate that contrary to *M. smegmatis,* induction of antisense of both *gyr*A and *gyr*B in *M. tuberculosis* resulted in greater than 3 log_10_ reduction in cfu. Thus, validating our general hypothesis that both *gyr*A and *gyr*B were indeed bactericidal targets. Interestingly the kill kinetics of moxifloxacin even at its MBC of 1 ug/ml, is much rapid than the gyrase antisense (Figure 8). This probably is due to the fact that the drug not only inactivates gyrase, but also traps the enzyme DNA complex setting up a down stream complex cascade resulting in generation of oxidative stress leading to a faster cell death-a process that is probably not initiated in the simple downregulation or inhibition of gyrase.

### Conditional KD of *inh*A, *emb*B, *rpo*B, *rpo*C, *rpl*J and *rps*L

The standard antitubercular therapy includes frontline drug such as isoniazid, rifampicin and ethambutol, which targets the genes *inh*A, *rpo*B subunit of RNA polymerase and *emb*B. In addition to Streptomycin, the antibiotic Linezolid and some modified macrolides has shown promise and may become a part of second line anti-tubercular therapy. These molecules elicit their antibiotic response by binding to 30 S and 50 S ribosomal subunits. In order to delineate whether these compounds (rifampicin, isoniazid, ethambutol and streptomycin) elicit their efficacy by inhibiting the enzyme in-vivo or is a result of some unknown feature of the compounds, full-length genes *rpo*B, *rpo*C, *inh*A, *emb*B, *rpl*J and *rps*L were cloned in antisense in the vector pAZI9018b.

**Figure 9:**
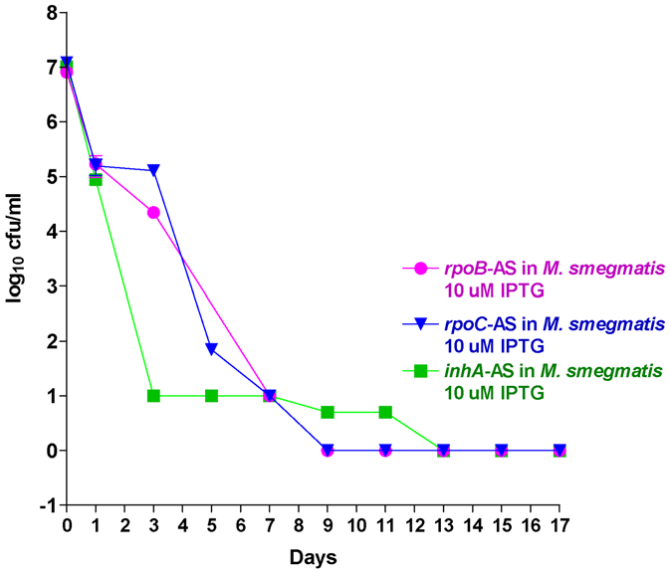
Survival kinetics of *M.smegmatis* (with *M. tuberculosis inh*A-AS plasmids individually). Induction at 10 uM IPTG concentration. *Inh*A-AS shows >3 log10 (bactericidal target) cfu reduction in *M. smegmatis*.

**Figure 10:**
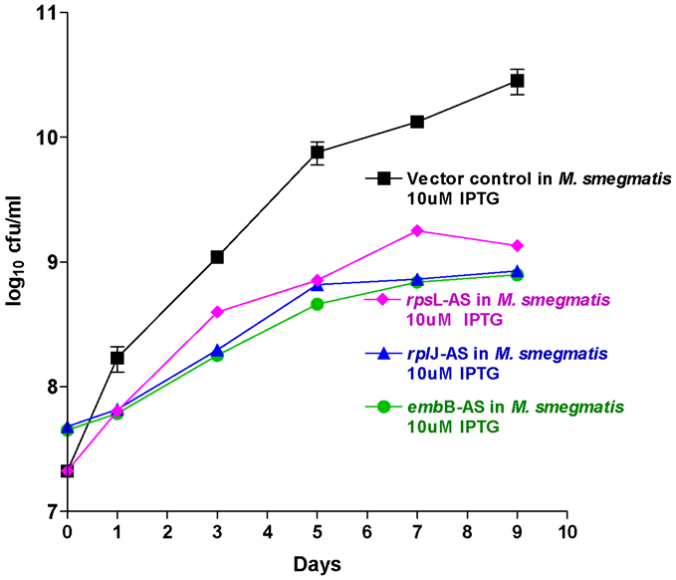
Survival kinetics of *M. smegmatis* (with *M. tuberculosis embB-*AS, *rps*L-AS & *rpl*J-AS plasmids). Inducing at 10 uM IPTG concentration. All of these antisense genes show a lesser growth rate as compared to the control, thus are bacteriostatic targets.

The resulting transformants were monitored by survival kinetics following induction. The results indicate that the knockdown of the genes *inh*A, *rpo*B and *rpo*C resulted in a gradual death of the bacteria indicating that these targets were indeed bactericidal and in line with our current thinking about their worth as validated anti-tubercular targets (Figure 9). In contrast, down regulation of embB only shows a minor growth inhibition compared to the wild type (Figure 10). These results are not surprising. Although the molecular basis of action of the pro-drug isoniazid is complex, it is proven that inhA is its primary target [20]. It could also be argued that through the inhibition of *inh*A, in addition to inhibition of mycolic acid biosynthesis it could also alter the NAD+/NADH ratio, which is a signal of cellular death. Recent results indicate that compounds that directly inhibit *inh*A are also efficacious [21]. Thus we have a strong evidence that knockdown of *inh*A could be bactericidal. The antisense inhibition of *rpo*B and *rpo*C in *M. smegmatis* causes rapid cidality (Figure 9). A similar picture was also observed in *M. tuberculosis* (data not shown.). In contrast the connection between *emb*B and ethambutol is being questioned. Recent clinical data indicate a lack of correlation between *embB* mutation and Ethambutol MIC in *M. tuberculosis* [22]. In addition it is seen that although the genes *emb*A and *emb*B are essential in *M. tuberculosis*, the deletion of these genes is viable in *M. smegmatis* although the growth rate is affected [23]. Our experiment evaluates the survival of *M. smegmatis* following the expression of *emb*B antisense, and the results are in accordance to the *emb*B knockout characteristics [23]. The down regulation of the ribosomal genes *rpl*J and *rps*L also did not elicit any major growth modulation; the survival kinetics indicated that it could be classified as “barely bacteriostatic” (Figure 10). This result is in consonance with the recent finding that of the several classes of antibiotics that target the ribosome only the aminoglycosides, kanamycin and gentamycin are bactericidal as it induces mistranslation and misfolding of membrane proteins resulting in generation of oxidative stress the causative agent for cidality [24]. In contrast, antibiotics like spectinomycin (ribosome inhibitors) are bacteriostatic as it only prevents translation step (and does not induce mistranslation) effectively analogous to the antisense expression of *rpl*J and *rps*L construct.

### Specificity of antisense inhibition: Reversible KD of *ilv*B

**Figure 11:**
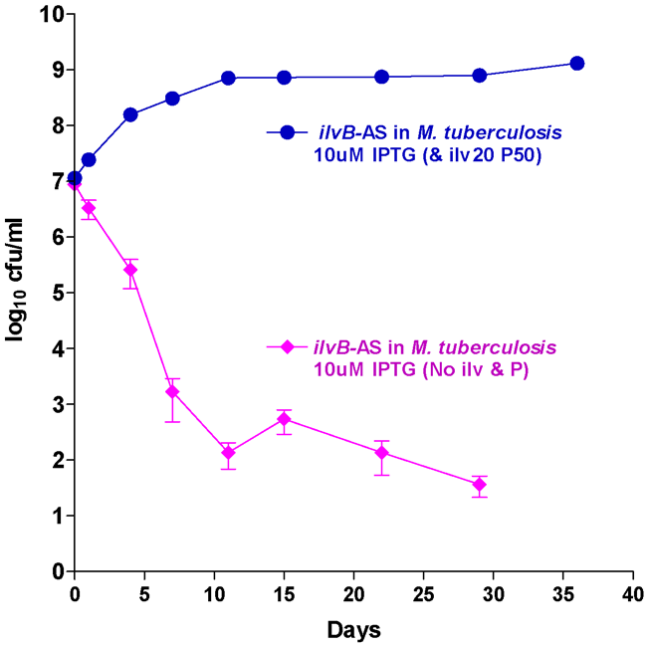
Survival kinetics of *M. tuberculosis* (with *M. tuberculosis ilvB*-AS plasmid). Induction at 10 uM IPTG concentration. The transformants grown in the in the absence of the supplements showed a strong antisense effect with >3 log10 cfu reduction (bactericidal target). This effect was reversed when grown in the presence of supplements (isoleucine, leucine, valine and pantothenate).

Though antisense effect shows profound effect with some genes, the question might remain on the specificity of antisense driven survival kinetics. The simplest way to test specificity of antisense effect is to monitor the survival kinetics of a known auxotropic target. The gene *ilv*B (Rv3003c) is involved in the synthesis of branched chain amino acids, isoleucine, leucine and valine and also pantothenate. The gene is essential when the bacterium grows in minimal media, which is not the case when grown in presence of the aminoacids and pantothenate. The survival kinetics (Figure 11) of antisense of *ilv*B in absence and presence of amino acids shows that the antisense effect can be completely reversed in presence of the amino acids. This result clearly indicates the specificity of gene knockdown by antisense expression. In order to characterize the behaviour of the local transcriptome following the antisense expression of *ilv*B, qRTPCR of the genes in the branched amino acid pathway was carried out. The results indicate that as predicted the entire pathway was down regulated (Table 1), thus additionally suggesting the specificity of antisense inhibition. Though the antisense was targeted against *ilv*B gene, only 2-fold downregulation of the *ilv*B, *ilv*N and *ilv*C transcripts were seen. These three genes are in an operon and form a polycistronic message. In contrast the other genes in the pathway were down regulated to a larger extent. This apparent variation was clarified by the observation that by using thermostable reverse transcriptase in the RTPCR we were capturing both the mRNA and the mRNA-antisense hybrid (data not shown). The transcriptional attenuation of *ilv* regulon in prokaryotes is a concerted phenomenon [25]. Hence we have the down regulation of entire pathway following antisense inhibition.

**Table 1:** Fold down regulation of genes in the branched chain amino-acid pathway in the *ilv*B antisense *M. tuberculosis* strain.

Gene	Mean	StDev
***ilvB***	2	0.3
***ilvA***	4.76	1.04
***ilvC***	2.21	0.4
***ilvD***	12.12	1.94
***ilvE***	14.66	1.97
***ilvG***	24.52	2.06
***ilvN***	2	0.31
***LeuA***	7.69	1.33
***LeuB***	39.36	4.21
***rpoB***	1.02	0.02

## Discussion

Discerning whether an *in-vivo* inhibition of a target enzyme would lead to downstream growth characteristics that range from bactericidal to bacteriostatic to ineffective has always been evaluated by compounds that specifically inhibit the target enzyme. However for novel target driven anti-infective drug discovery, the paucity of this relevant information leads to an expensive alternative in evaluating the target through high throughput screening (HTS). However the success in this process as measured by identification lead molecules that kill the bacteria through specific mechanism of action is minimal [2]. We have described a simpler alternative that utilizes inducible vector system to characterize novel targets. One of the key assumptions of this exercise is that specific in-vivo inhibition of a target enzyme by its inhibitor is equivalent to down regulation of the target enzyme by antisense expression. This assumption is logically tenable as a 50 % inhibition of enzyme activity can be thought either as a case where 50% of the enzyme molecules are fully active while the rest is inactive to the alternative where every molecule is active only half the time. The former simulates the case of inhibition by an irreversible inhibitor while the later is similar to the case of reversible one. In our current model of specific antisense expression, it is the number of active enzyme molecules that are reduced and hence the growth characteristics as monitored by survival kinetics would simulate an effective compound inhibiting the target enzyme.

Inducible systems are powerful tools for studying gene function and for the validation of drug targets in bacteria. However few regulated systems are available for mycobacterium gene expression [26], [17], [27]. The characteristics governing an ideal inducible promoter system are- 1: there should be little or no expression of the target gene in the absence of an inducer, 2: the strength of the promoter should be moderate enough to give an appreciable window within the working range of the inducer, 3: the non-metabolizable inducer should not be required to be present at toxic levels, 4: it should regulate in a dose dependent manner, 5: in case of the studies in an intracellular pathogen, it should allow the regulatable expression under in-vivo conditions. However, the crucial feature of an inducible system should be that the inducer enters the bacteria by diffusion-controlled mechanism. For inducer entering the cell via active transport there is an “all or none” phenomenon. Thus at sub-saturating concentration of the inducer, some cells are fully induced while majority remains uninduced [11]. Under this scenario it would be difficult to interpret survival kinetics. The linearity of induction as exemplified in the β-galactosidase assay provides a clue that in mycobacteria, IPTG enters the cell via passive diffusion.

Our results broadly suffice the above-mentioned characteristics. We have also seen the possibility of system to operate when the bacteria is present in intercellular conditions, (data not shown). In order to achieve an ideal expression system described above for mycobacteria, we have used *SigA* dependent promoters that operate under normal growth conditions. Using the consensus *Sig*A motif as predicted by Unniraman *et al*. [13] we showed that the well-studied *E. coli* pTrc promoter was also effective in mycobacterium. The control of inducible expression is a direct interplay between the repressor concentration, effector concentration and the binding constant of the operator. This interplay had been modeled by Yagil and Yagil [12]. We have used the simple time independent formulation to simulate a condition that gives a linear dose response. It is seen that for a given pair of repressor and operator there are multiple ways to alter the nature of dose response. To tighten the control one could increase the concentration of the repressor or increase the number of operator molecules upstream of the gene. As described earlier we have used a moderate strength constitutive mycobacterial promoter to express sufficient *Lac*I that gives a linear dose response with tight control. However compared to the induction profile of protein expression as in case of β-galactosidase where we have an excellent window of expression, the dose dependence for survival kinetics with antisense expression has a much smaller window (data not shown). In case of former the induction time is four hours whereas the time for monitoring survival kinetics is in days. The lack of a decent induction window in case of survival kinetics can be understood in light of product accumulation. The antisense RNA expression in absence of inducer, which is negligible in a four-hour time span, might become significant when the induction time is in days due to accumulation of product. This has been observed with β−galactosidase expression. It could be similar with antisense expression.

The antisense induction of various targets for standard anti-tubercular drugs like *inh*A for isoniazid, *rpo*B and C for rifampicin, and *gyr*A/B for flouroquinolones were shown to be bactericidal. In contrast targets like *Fts*Z showed only a moderate growth inhibition. That the targets *inh*A and *emb*B differed so dramatically in their survival kinetics is probably explained by their contrasting essentiality in *M. smegmatis*. We have also monitored antisense *inh*A survival kinetics in *M. tuberculosis*. The results indicate that the target is in the bactericidal zone (data not shown). The antisense expression of ribosomal RNA genes like *rpl*J and *rps*L produced “barely bacteriostatic” effect. This is probably due to long half-life of the ribosome and the fact that antibiotics that target ribosome affect protein folding rather than ribosome recycling [28]. Additionally it has been recently shown that among the antibiotics that target ribosome, aminoglycosides like kanamycin and gentamycin are bactericidal because they induce membrane proteins to mistranslate and hence misfold in the periplasmic space leading to generation of free radicals [24]. In contrast, some ribosome inhibitors like spectinomycin are bacteriostatic, as they do not initiate mistranslation. It would thus be logical to speculate that antisense *rpl*J and *rps*L function as simple ribosome inhibitors and thus do not induce cidality. Additionally it should also be noted that compared to mRNA, ribosomal RNA are much more stable and has a longer half life [29]. It is also structurally more robust than mRNA and its concentration is also higher than a specific mRNA. All of these factors adding up and blunting the effect of antisense *rpl*J and *rps*L.

Of these, the results for *gyr*B in *M. smegmatis* in a way validate the whole approach. While designing the experiment we had inadvertently assumed *gyr*B to be essential in *M. smegmatis*. However when the results indicated that antisense of *gyr*B did not show any bactericidal effect in its survival kinetics we realized the presence of an orphan *gyr*B earlier identified [19]. In order to prove the validity of this argument we did evaluate the survival kinetics of antisense *gyr*A and *gyr*B in *M. tuberculosis*. The result indicated that the down regulation of both these genes were bactericidal as there were no homologs of the gyrase genes present in this species. It is also seen that moxifloxacin induced killing is faster than gyrase antisense, thus indicating the possibility with flouroquinolones the inhibition of gyrase is complex and the mechanism of cell death is mainly related due to its downstream effects, a fact that is well known [30], [31].

That these results were specific for the genes concerned and not due to any off target effect were further validated by the survival kinetics of *ilv*B antisense expression. The bactericidal effect was totally reversed in presence of isoleucine; leucine, valine and pantothenate indicated the specificity of antisense induction. It was also seen that following *ilv*B antisense expression, the branched chain amino acid biosynthesis transcriptome was specifically down regulated.

It is becoming gradually accepted that bactericidal drug targets are better targets for anti-mycobacterial drug discovery. It has recently been demonstrated that at least in *E. coli* and *S. aureus,* bactericidal action of antibiotics is due to the induction of hydroxyl radicals, while bacteriostatic antibiotics do not produce them [31]. If this method of cellular death is true in case of slowly growing mycobacteria then it would be interesting to see whether knockdown of bactericidal targets also produce the same radicals. However generalization across species might sometimes be fraught with the danger of oversimplification. The antibiotic Rifampicin, which is bactericidal in *M. tuberculosis*, is bacteriostatic in *E. coli* [31]. We feel that the present technology will aid in identifying novel targets for *M. tuberculosis* whose inhibition would lead to the death of the bacteria. Finally one of the interesting points raised by these experiments concerns the value of using *M. smegmatis* as a model system for *M. tuberculosis*. It seems that for genes, which are present as a single copy in both the organisms, and for pathways where there is a one to one correspondence, the answers to biological question will be qualitatively similar. It might also be appreciated that if the time axis of the survival kinetics is normalized to number of generations instead of number of days, both the mycobacterium species behave in a somewhat similar fashion.

One of the major factors that drive the consistency of antisense expression in slow growing mycobacteria is the stability of the shuttle vector under induced conditions for at least eight weeks. We had tested the stability of the plasmid by growing the bacteria in absence of Hygromycin and measuring colony forming units (cfu's) in presence and absence of the antibiotic marker. The results indicate (data not shown) that the plasmid pAZI9018b was stable during the period of induction.

## Materials and Methods

### Bacterial strains, media, competent cells and transformations

*E. coli* MOS Blue cells {F'*end*A1 *hsd*R17 (r_K_^−^ m_K_^+^), *sup*E44 *thi*-1 *rec*A1 *gyr*A96 *rel*A1 *lac* [F' *lac*I^q^ZΔM15 *pro*AB^+^ *Tn*10 (Tet^R^)]} (Amersham) were used for general cloning and propagation host. *E. coli* Able K {*E. coli* C *lac* (*Lac*Zω^–^) [Kan^r^ *Mcr*A^–^ *Mcr*CB^–^ *Mcr*F^–^ *Mrr* ^–^ *Hsd*R (r_K_^−^m_K_^−^)] [F' *pro*AB *lac*I^q^ZΔM15 *Tn*10 (Tet^r^)]} (Table 2) from Stratagene replaced *E. coli* MOS Blue cells as the propagation host for pAZI9018b plasmid; as the use of this strain reduces the copy number by ∼10 times which to a large extent alleviates the problem of plasmid instability. Electrocompetent *E. coli* cells were prepared by growing the cells in SOB medium to an O.D._600 nm_ of 0.7–0.8, chilled on ice, centrifuged at 1500 ×g, thrice washed with equal culture volume chilled 10% glycerol, resuspended in 10% glycerol to a final O.D._600 nm_ = 100, flash frozen in dry ice and stored at −70°C.

**Table 2:** Bacterial strains and plasmids used in this study.

Strains	Description	Source or Reference
*E. coli* DH5α	Competent cells	AZI Collection
*E. coli* pMOS Blue	Competent cells	Amersham Biosciences
*E. coli* AbleK	Competent cells	Stratagene
*M. smegmatis* mc^2^ 155	Gene expression and survival kinetics studies	AZI collection
*M. tuberculosis* H37Rv ATCC 27294	Gene expression and survival kinetics studies	ATCC
**Plasmids**	**Description**	**Source or Reference**
pSMT3	*E. coli*-mycobacterial shuttle vector	Kindly provided by Dr. Neil Stoker, London School of Tropical Medicine and Hygiene,UK.
pEU720	for β-galactosidase gene	Kindly provided by Dr. Scott, Emory University ^7^
pTrc 99A	for pTrc Promoter	Pharmacia

*Mycobacterium smegmatis* mc^2^155 was maintained in 7H9 broth (DIFCO) +0.15% Tween or 7H11 agar (DIFCO) supplemented with OADC. Similar conditions were maintained for slow growing mycobacteria *M. tuberculosis* H37Rv ATCC 27294. Cells were grown in the above medium and competent cells were prepared similar to *E. coli* MOS Blue cells with the variation that washing and suspension of the cells were done in 10% glycerol with 0.1% Tween. Three different independent transformations were performed. Transformations in *E. coli, Mycobacterium smegmatis* mc^2^155, and *M. tuberculosis* H37Rv ATCC 27294 were done at 4°C with common settings of 25 μF, 1000 Ω and 2.5 kV in 0.2 cm cuvette in a BIO-RAD Gene-Pulser® II electroporation unit. After pulsing, cells were resuspended in prewarmed respective media and recovered for two-generation times before plating on selective medium. Hygromycin (Roche) was always used at a concentration of 50 µg/ml. The plates were incubated for ∼30 generation time for each species. The transformants were picked up from the triplicate transformations in 7H9 broth (with supplements) containing 50 ug/ml hygromycin for mycobacteria and in LB in case of *E. coli*. The O.D._600 nm_ was adjusted to ∼0.1 and incubated for 24 hrs at 37°C. The cells were divided into 5 different aliquots and induced at 0, 1, 10, 100 and 1000 µM IPTG. The antisense effect was estimated by plating for the cfu enumeration on different generation times (*M. smegmatis*: Day 0, 1, 3, 5, 7 and for *M. tuberculosis*: Day 0, 1, 7, 14, 21, upto 63 days with a gap of 1 week).

### Plasmids and Enzymes

pSMT3 is a shuttle vector with *E. coli* pMB1 origin of replication (relaxed replication), a hygromycin resistance gene and the mycobacterial pAL5000 origin of replication (stringent replication with a copy number of 3–4). pEU720 [32] contained a promoterless *LacZ* gene flanked by multiple rrnB terminators. *LacI* gene was derived from p*Trc*99A (Pharmacia). DNA polymerase Phusion (Finnzymes) was used in all amplification reactions according to manufacturer's instructions supplemented with DMSO and Betaine. Other enzymes were either from NEB or Amersham Biosciences.

### Cloning of pAZI 9018b

The cloning was strategized (Figure 2) in a way so as to minimize the number of steps required and also to optimize the size of the final vector which would contain the following seven elements: 1) *LacZ* as the reporter gene, 2) a mycobacterial *sig*A promoter to drive this gene, 3) a lac operator controlling this promoter, 4) *LacI* gene driven by a constitutive mycobacterial (*sig*A) promoter, 5) *E. coli* origin of replication, 6) mycobacterial origin of replication and 7) hygromycin resistance gene. Constructs at different stages were named according to the elements present on them.

DNA fragment strictly encompassing the three elements *Ori*E, *Ori*M and hygromycin resistance gene [33] was amplified from pSMT3 using primers HYGR and MORR (Table 3), gel purified, digested with *Nhe*I and *Dpn*I, ligated and transformed in *E. coli* to get the plasmid p567. Next step was to insert the promoter-less reporter gene *LacZ* flanked by multiple transcriptional terminators so as to minimize any non-specific transcription. This fragment was amplified from pEU720 using primers PTLF2 and PTLR3 *Apa*I, gel purified, digested with *Mfe*I and *Apa*I and ligated to *Mfe*I and *Apa*I digested p567, followed by transformation in *E. coli* to get the plasmid p1567'. However, the pSMT3-derived fragment carried a pHsp60 promoter between *Ori*E and *Ori*M. To delete this region p1567' was digested with *Not*I and *Msc*I, completely filled in using Klenow (Exonuclease free), self-ligated and transformed in *E. coli* to get p1567.

**Table 3:** Primers used in the present study.

Primer	Sequence (5′-3′)
HYGR	CATGCTAGCTCAGGCGCCGGGGGCGGTGT
MORR	CATGCTAGCTAGAACAGCGGTGGATTGTC
PTLF2	CATCAATTGCGTCTTCAAGAATTAATTCCCAA
PTLR3APAI	CATGGGCCCCGCTGCCCGACTGCTTTC
LACO5A	AGCTATGTTGACATTTTTTTCAATATTTGTTATAATGTGTGGGGAATTGTGAGCGGATAACAATTCCCCTGCA
LACIPT150	CATAAGCTTGAATTCTTGACACTTTGCGACACGCTTTTATCATTTTCCGACATATAAGAAGGAGGATTCAGGGTGGTGAATGTGA
Ptrclaco rev	CATAAGCTTTCCTGTGTGAAATTGTT
FtsZR	ATTCCAACATATGATGACCCCC CCGCACAACTA
FtszF	AGGCCTAGGATCCTCAGCGGCGCATGAAGGGCG

Next step was to insert a promoter with a lac operator upstream of the *LacZ*. However, insertion of such a fragment in p1567 (see p123567 in Figure 2) in absence of any lac repressor resulted in severe plasmid instability and severe cell growth inhibition in *E. coli* MOS Blue, probably due to very high constitutive expression of *Lac*Z. Thus we decided to introduce a constitutive Lac repressor along with. This fragment, which would incorporate a *Lac*I gene driven by the constitutive promoter T150 and a Trc promoter with a lac operator upstream of *Lac*Z, was amplified from p*Trc*99A using primers PTRCLACOREV and LACIPT150, digested with *EcoR*I and *Hind*III and ligated to purified *Mfe*I and *Hind*III digested p1567 and transformed in *E. coli ABLE K* (Stratagene). The resulting transformants were stable. The final vector was designated as pAZI9018b.

### Validation of the inducible promoter system by β-galactosidase assay

The plasmid pAZI 9018b (Figure 3) was transformed into *E. coli ABLE K* for propagation and *M. smegmatis mc^2^ 155* for the β-galactosidase activity assay respectively. The *M. smegmatis* colonies were grown in 7H9 selective media containing hygromycin at 50 µg/ml concentration. Upon reaching an O.D._600 nm_ = 0.6, each set of culture was divided into 7 in a 24 well plate and were induced with 0, 1, 10, 100, and 1000 µM of IPTG (Sigma) respectively for 4 h at 37°C (for *M. smegmatis*). 500 µl of each induced culture was then lysed on ice by two 30-second cycles of sonication at 15 seconds interval. The β-galactosidase assay was done with 40 μl of lysate in 260 μl of the Z buffer [0.06 M Na_2_HPO_4_.7H_2_O, 0.04 M NaH_2_PO_4_.H_2_O, 0.01 M KCl, 0.001 M MgSO_4_, 0.05 M β-mercaptoethanol, pH 7.0 containing 1 mg/ml of ONPG as substrate at 37°C and the O.D._410 nm_ was monitored using Spectramax (Molecular Devices, SpectraMax Plus^384^ spectrophotometer). The assay was done in triplicate and each experiment was repeated at least twice.

### Validation of inducible system by antisense inhibition of essential genes *FtsZ, gyr*A, *gyr*B, *rpo*B, *rpo*C, *inh*A, *emb*B, *rps*L and *rpl*J from *M. tuberculosis* in *M. smegmatis*

Full-length *M. tuberculosis FtsZ, gyr*A, *gyr*B, *rpo*B, *rpo*C, *inh*A, *emb*B, *rps*L and *rpl*J genes were PCR amplified from genomic DNA using the forward and reverse primers (*Fts*ZR, *Fts*ZF, *gyr*AR, *gyr*AF, *gyr*BR, *gyr*BF, *rpo*BR, *rpo*BF, *rpo*CF, *rpo*CR, *inh*AR, *inh*AF, *emb*BF, *emb*BR, *rps*LF, *rps*LR, *rpl*JF and *rpl*JR) and cloned into *Nde*I and *BamH*I (or *Xba*I) sites of the vector pAZI9018b. These constructs (*FtsZ*-pAZI 9019, *gyr*A-pAZI 9400, *gyr*B-pAZI 9401, *rpo*B-pAZI 9402, *rpo*C-pAZI 9403, *inh*A-pAZI 9404, *emb*B-pAZI 9435, *rps*L-pAZI 9436 and *rpl*J-pAZI9437) that had the target genes in the antisense orientation were transformed into *M. smegmatis* competent cells by electroporation. The colonies were picked up and the cultures were grown in 7H9 broth. The growth was very slow. For the induction experiment, the culture had to be concentrated to match the O.D._600 nm_ of the control culture. The cells were allowed to undergo four generations in the induced form. In the case of *Fts*Z culture, the morphology was examined by light microscopy (1000X) under phase contrast conditions (Figure 4).

### Validation of inducible system by antisense inhibition of essential genes: *M. tuberculosis gyr*A and *gyr*B in *M. tuberculosis*

The antisense constructs of full length *gyr*A, *gyr*B, and *inh*A genes were transformed in *M. tuberculosis* competent cells at room temperature with the similar BIORAD electroporator settings as for *M. smegmatis*. The transformants were picked up from the triplicate transformations in 7H9 broth (with supplements) containing 50 ug/ml hygromycin. The O.D._600 nm_ was adjusted to ∼0.1 and incubated for 24 hrs at 37°C. The cells were divided and induced at 0, 1, 10, 100 and 1000 µM IPTG. The effect of antisense was measured by plating for the cfu enumeration on different days. All experiments pertaining to *M. tuberculosis* were in triplicate, whereas those with *M. smegmatis* are repeated twice.

### Specificity of anti-sense inhibition: Reversible survival kinetics of *ilv*B

Full-length *M. tuberculosis ilvB* gene was PCR amplified from genomic DNA using the forward and reverse primers and cloned into *Nde*I and *BamH*I site of the vector pAZI9018b. The construct (*ilv*B-pAZI9406) that had the target gene in the antisense orientation was transformed into *M. tuberculosis* competent cells by electroporation.

The transformants were picked up and O.D._600 nm_ was adjusted to 0.1 with approximately 10^7^ cells/ml. These were grown in the presence and absence of ilv20 (isoleucine, leucine and valine, each @ 20 ug/ml conc. and pantothenate @ 50 ug/ml conc.). The cfu enumeration was performed on 1, 3, 5, 7, 14 and weekly upto about 63 days; in the presence and absence of IPTG (inducer @ 10 uM conc.). The survivors' cfus were plotted against the number of days.

### Branched chain amino acid transcriptome analysis following antisense expression of *ilv*B gene in *M. tuberculosis*

*M. tuberculosis* cells containing *ilvB* antisense plasmid were grown as log phase culture in the presence of ilvP (isoleucine, leucine and valine, each at 20 ug/ml conc. and pantothenate at 50 ug/ml conc. The inducer IPTG was at 100 uM conc.). Wild type (WT) *M. tuberculosis* culture was also grown in a parallel log phase culture without the supplements. One ml of Trizol® was added to the cell pellets (from 5 ml culture) to stabilize and arrest the mRNA. Cells were disrupted by bead beating using 0.1 mm diameter zirconium beads (Biospec), followed by a 5 min centrifugation at 14,000 g. Total RNA was isolated by chloroform treatment and precipitated by using isopropanol and centrifuged 14,000 g for 20 min. The RNA pellet was washed with 70% ethanol, repelleted at 14,000 g for 10 min. RNA samples were further treated by DNAse I treatment (Ambion Cat# 2222-DNAse) for 30 min. at 37°C to remove any residual DNA contamination. The sample was then purified using RNEasy mini kit, Qiagen).

RNA concentration was estimated using nanodrop spectrophotometer. An aliquot 100 ng of RNA was used for each WT and *ilv*B antisense recombinant Mtu for cDNA synthesis and RTPCR. A total of 11 genes were selected from the branched chain amino acid pathway to see the after effect of *ilvB* gene down regulation on the other genes in the same pathway. *Rpo*B (Rv0667) was selected as the house-keeping gene. RTPCR was performed in quadruplicate using Qiagen (single tube) RTPCR kit using SYBR Green chemistry. Real-time PCR (Mx3000P Stratagene) conditions include initial denaturation at 95°C for 15 min, followed by 40 cycles of denaturation at 94°C for 30 sec, annealing and extension at 59°C for 30 sec. Reverse and forward primers used are listed below in Table 4. The fold down regulation of the expression of the genes in the *ilv*B antisense strain was calculated using the equations described below. The normalization factor, i.e. the level of expression of a gene compared to *rpo*B was first estimated in the WT cells.



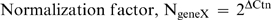
(1)Where 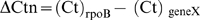


Ct defined as the threshold cycle for each gene. The fold down regulation, 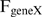 for each gene in the *ilv*B antisense strain was then calculated using the equation 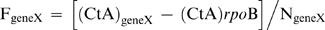
(2)Where CtA is the corresponding threshold cycle of the gene in the antisense strain.

**Table 4:** Primers used for transcriptome analysis.

S.No.	Gene primers
1	*ilv*B (F) 5′- GCGCAGTTCATCAGATACGA-3′
2	*ilv*B (R) 5′- CTGGTTGGTCATCTGGAAGC -3′
3	*ilv*A (F) 5′- ACTATCTGGGGCAGACATCG -3′
4	*ilv*A (R) 5′- GCTTATAAGAGCGCACCGTC -3′
5	*ilv*C (F) 5′- AGGTTGGTGTGATCGGCTAC -3′
6	*ilv*C (R) 5′- CTTGAGGTTGGGTTCGATGT-3′
7	*ilv*D (F) 5′- TGTTCTACTGCTGACCGACG -3′
8	*ilv*D (R) 5′- ACTGAAATCCTGTTGTCGGG -3′
9	*ilv*E (F) 5′- CTCCCTTCAATTCACGGTGT -3′
10	*ilv*E (R) 5′- ATCGGGCCATAAGGGATTAC-3′
11	*ilv*G (F) 5′- ATCGACAGCTATCTGCCAGG -3′
12	*ilv*G (R) 5′- TTGTTGCCGATCACTGACAC -3′
13	*ilv*N (F) 5′- GATCACCAAGCAGCTCAACA -3′
14	*ilv*N (R) 5′- CTCTAACTTGCCGCGGTTAC -3′
15	*Leu*A (F) 5′- AAGGAGATTGAGGTGGGGTT -3′
16	*Leu*A (R) 5′- TTGTAGAAGTGCACGATGGC -3′
17	*Leu*B (F) 5′- CATTGGTGCACAAAACGAAC-3′
18	*Leu*B (R) 5′- AACAGGTTGTCGGTGACGAT-3′
19	*rpo*B (F) 5′- CCTGGAAGAGGTGCTCTACG-3′
20	*rpo*B (R) 5′- TGTCCTTGTCTTTGCACTCG-3′

## Supporting Information

File S1 (0.03 MB DOC)
