# Supplementary material for: Delineating Bacteriostatic and Bactericidal Targets in Mycobacteria Using IPTG Inducible Antisense Expression
Source: PLoS One. 2009 Jun 15;4(6):e5923. doi: 10.1371/journal.pone.0005923 (PMC2691988; doi:10.1371/journal.pone.0005923)
Supplement: File S1 — (0.03 MB DOC) [file pone.0005923.s001.doc]

**Supplementary information:**

Let *R*, *O* and *E* be the intracellular active concentration of *Lac* repressor (*Lac*I), *Lac* operator (*Lac*O) and the effector IPTG respectively and n be the effective number of IPTG molecules required to bind a single *Lac*I tetramer and abolish its affinity to the operator DNA. These interactions can be represented as two competing equilibria given by

n.E + R REn ….(1)

K1

K2

O + R OR … .(2)

Where REn is the complex of the repressor with n IPTG molecules, and OR is the operator repressor complex. The equilibrium constants K1 and K2 are given by

K1 =

[REn]

[R].[E]^n^

K2 =

[OR]

[O].[R]

It has been shown by Yildirim and McKay [1] that if these two reactions are assumed to be of the above forms and if the amount of repressor bound to operator is small compared to the total amount repressor present in the cell (*R*_tot_), then the fraction of operators defined as ƒ(E), not bound to repressor and hence capable of synthesizing mRNA, is given by

……(3)

ƒ(E) =

1+ K_1_.E^n^

K+ K_1_.E^n^

where K= 1+K_2_.*R*tot …… (4)

It follows from equation 3, that the maximum repression would be in the absence of IPTG i.e. when *E*=0. However even at this condition since ƒ(E) is not equal to zero, there would be some leaky expression that is proportional to 1/*K*. This implies that the degree of leaky expression can be decreased by increasing the magnitude of *K*. In accordance to equation 4 this can be achieved, either by increasing total amount of intracellular repressor (*R*_tot_) or by increasing the association constant (*K*_2_) of the binding of repressor with operator. Increasing the *Lac*I expression can be accomplished using a stronger constitutive promoter, while increasing the affinity between the repressor and the operator can be achieved by using *Lac*I or a *Lac*O mutant [2]. As an alternate strategy for decreasing leaky expression one could use multiple (p) operators in tandem. Under such a scheme, if the interaction between each repressor and operator are independent, then the fraction of operators that free of repressors are given by

[

K + K_1_ E^n^

]

ƒ(E) =

1+ K_1_ E^n^

p

………(5)

Therefore, in the absence of any IPTG, the fraction of operators not bound by repressor would be proportional to [1/*K*]^p^. In this case the leaky expression will be exponentially reduced.

###### References:

1. Yildirim N and Mackey M C (2003) Feedback regulation in the lactose operon: A mathematical modeling study and comparison with experimental data. Biophys J 84:2841–2851.
2. Falcon C M and Matthews KS (2000) Operator DNA sequence variation enhances high affinity binding by Hinge Helix mutants of Lactose repressor protein. Biochem 39: 11074-11083.
